# The Oncolytic Virus MG1 Targets and Eliminates Cells Latently Infected With HIV-1: Implications for an HIV Cure

**DOI:** 10.1093/infdis/jix639

**Published:** 2017-12-08

**Authors:** Nischal Ranganath, Teslin S Sandstrom, Stephanie C Burke Schinkel, Sandra C Côté, Jonathan B Angel

**Affiliations:** 1 Biochemistry, Microbiology, and Immunology, University of Ottawa, Ottawa, Canada; 2 Ottawa Hospital Research Institute, Ottawa, Canada; 3 Department of Medicine, The Ottawa Hospital, Ottawa, Canada

**Keywords:** HIV-1 latency, HIV-1 reservoir, CD4^+^ T-cell, oncolytic virus, Maraba virus, MG1

## Abstract

Cells latently infected with human immunodeficiency virus (HIV) evade immune- and drug-mediated clearance. These cells harbor intracellular signaling defects, including impairment of the antiviral type I interferon response. Such defects have also been observed in several cancers and have been exploited for the development of therapeutic oncolytic viruses, including the recombinant Maraba virus (MG1). We therefore hypothesized that MG1 would infect and eliminate cells latently infected with HIV-1, while sparing healthy uninfected cells. Preferential infection and elimination by MG1 was first demonstrated in cell lines latently infected with HIV-1. Following this, a reduction in HIV-1 DNA and inducible HIV-1 replication was observed following MG1 infection of latently infected, resting CD4^+^ T cells generated using an in vitro model of latency. Last, MG1 infection resulted in a reduction in HIV-1 DNA and inducible HIV-1 replication in memory CD4^+^ T cells isolated from effectively treated, HIV-1–infected individuals. Our results therefore highlight a novel approach to eliminate the latent HIV-1 reservoir.

## Teaser

Latent human immunodeficiency virus type 1 (HIV-1) infection represents a significant barrier to the development of an HIV-1 cure. This study demonstrates that latently infected cells are eradicated by the cancer-killing virus MG1, suggesting a novel strategy by which to eliminate the HIV-1 reservoir.

Latent human immunodeficiency virus type 1 (HIV-1) is maintained within long-lived cellular reservoirs as replication-competent, proviral DNA [1, 2]. These reservoirs are composed primarily of resting memory CD4^+^ T cells, which evade immune- and drug-mediated clearance and serve as a source of infectious virus upon treatment cessation [3, 4]. Current HIV cure strategies are therefore geared toward the elimination of these cells.

Detailed characterization of HIV latency remains a challenge. The absence of reliable phenotypic markers and the overall rarity of cells latently infected with HIV-1 in circulation prevent the enrichment of these cells ex vivo [2, 3]. Consequently, a number of primary cell models have been established [5] and have shown to be useful in the screening of various pharmacological agents intended to reactivate and subsequently eradicate latent provirus [6, 7]. These models are characterized by extremely low frequencies of cells latently infected with HIV-1, making them poorly suited for investigating biological mechanisms of reactivation and killing. As such, cell line models have been used to investigate important differences between cells latently infected with HIV-1 and cells that are uninfected, including activation of cell death pathways upon stimulation [8] and impairment of antiviral responses mediated by type I interferon (IFN-I) [9].

The IFN-I response represents the first-line host defense against viral infections. HIV-1 has evolved countermeasures to escape IFN-I–mediated immune control during productive infection, including degradation of pattern-recognition receptors [10, 11], inhibition of IFN regulatory factors [12], and impairment of antiretroviral restriction factors [13, 14]. While the ability to investigate IFN-I signaling defects during latent infection of primary cells remains a challenge, we have recently demonstrated that the induction of various IFN-stimulated genes following IFN-α or polyinosinic:polycytidylic acid stimulation is impaired in cell lines latently infected with HIV-1 [9]. Impaired IFN-I signaling in cell lines latently infected with HIV-1 may therefore represent a target for the design of therapeutic strategies intended to eliminate major HIV-1 reservoirs.

Interestingly, IFN-I signaling defects in human cancers have led to the identification of several oncolytic rhabdoviruses capable of eradicating tumors cells [15, 16]. In particular, the genetically engineered Maraba virus (MG1) has been shown to target IFN-I–defective tumors in vivo with minimal toxicity [17, 18]. Given that IFN-I signaling defects have been observed during HIV-1 infection and, importantly, in latently infected cell lines, we hypothesized that cells latently infected with HIV-1 would be more susceptible to MG1 infection and killing than HIV-uninfected bystander cells.

## METHODS

### Reagents

Recombinant human chemokine (C-C motif) ligand 19 (CCL19) was purchased from R&D Systems (Minneapolis, MN), and raltegravir (RAL) was obtained from Santa Cruz Biotechnology (Dallas, TX). Phytohemagglutinin (PHA) was obtained from Sigma-Aldrich (Oakville, Canada), recombinant human interleukin (IL-2) was obtained from Cell Sciences (Canton, MA), and LEA- purified anti-human CD3 (OKT3 clone) and anti-human CD28 (CD28.2 clone) were obtained from BioLegend (San Diego, CA).

### Cell Lines and Cell Culture

U937 (CRL-1593.2), HL60 (CCL-240), Vero (CCL-81), and 293T (CRL-3216) cell lines were obtained through American Type Culture Collection. OM10.1 cells from Dr Salvatore Butera [19] and U1 cells from Dr Thomas Folks [20] were obtained through the National Institutes of Health (NIH) AIDS Reagent Program, Division of AIDS, National Institute of Allergy and Infectious Diseases, NIH. HIV latency and capacity for reactivation was confirmed in OM10.1 cells and U1 cells by measuring p24 release, using an enzyme-linked immunosorbent assay (ELISA; performed at the National Cancer Institute at Frederick AIDS and Cancer Virus Program). U937, U1, and OM10.1 cells were cultured in Roswell Park Memorial Institute 1640 medium supplemented with 10% heat-inactivated fetal bovine serum (FBS) and PSG (penicillin [100 U/mL], streptomycin [100 μg/mL], and L-glutamine [2 mM]; RP10 medium; all from ThermoFisher Scientific, Waltham, MA). HL60 cells were cultured in Iscove’s modified Dulbecco’s medium (IMDM; ThermoFisher Scientific) with 20% FBS and PSG. Vero cells and 293T cells were cultured in Dulbecco’s modified Eagle’s medium (ThermoFisher Scientific) with 10% FBS and PSG.

### Production of HIV-1 Stocks

HIV-1 stocks were produced by calcium-phosphate transfection (Promega, Madison, WI) of 293T cells, using 20 μg of pNL4-3 [21]. Virus preparations were filtered through a 0.2-μm polyvinylidene fluoride filter and quantified by p24 ELISA. Virus was then amplified on CD8-depleted peripheral blood mononuclear cells (PBMCs). Briefly, CD8-depleted PBMCs were isolated from healthy donors, using the EasySep Human CD8 Positive Selection Kit (Stemcell), and were activated in RP10 medium with PHA (5 μg/mL) and IL-2 (30 U/mL) for 3 days. Activated CD8-depleted PBMCs were then infected with HIV_NL4.3_ stocks, and 2 feeder cycles of activated, CD8-depleted PBMCs were added 7 and 10 days after infection. Culture supernatant was harvested 14 days after infection, and HIV_NL4.3_ virus preparations were filtered and quantified by p24 ELISA.

### Production of MG1 Stocks

Green fluorescent protein (GFP)–expressing recombinant MG1 was obtained from John Bell and Dave Stojdl and propagated in Vero cells as described previously [16, 17]. Viruses were obtained from cell supernatants and titrated on Vero cells by plaque assay.

UV light–based inactivation of virus was performed as previously described [22]. Briefly, MG1 stock was diluted to 1 × 10^9^ plaque-forming units/mL in phosphate-buffered saline (PBS) and irradiated by UV-C light at 120 mJ/cm^2^ for 2 minutes, using the Spectrolinker XL-1000 UV crosslinker (Spectronics, Westbury, NY). Confirmation of virus inactivation was assessed by infection of Vero cells followed by quantification of infection and viability by flow cytometry.

### Participants Infected With HIV and Receiving Combination Antiretroviral Therapy (cART)

HIV-infected individuals followed at the Immunodeficiency Clinic of the Ottawa Hospital were selected on the basis of sustained suppression of plasma viral load (<40 copies/mL for >6 months) during cART and CD4^+^ T-cell counts of >400 cells/μL.

### Isolation and Culture of Resting CD4^+^ T Cells From HIV-Seronegative Donors and Memory CD4^+^ T Cells From HIV-Infected cART Recipients

After informed consent was obtained, blood specimens were collected in heparinized syringes. PBMCs were isolated by Lymphoprep (Stemcell, Vancouver, Canada) density gradient separation. CD4^+^ T cells were purified with the EasySep Human CD4^+^ T-cell enrichment kit (Stemcell). Resting CD4^+^ T cells were enriched using mouse immunoglobulin G_1_ monoclonal anti-CD69 (clone FN50; BD Pharmingen, San Jose, CA) and anti-HLA-DR (Clone L203; R&D Systems, Minneapolis, MN) antibodies, using the EasySep Human Do-It-Yourself selection kit (Stemcell). Memory CD4^+^ T cells, defined as CD45RA^neg^CD45RO^+^CD4^+^ cells, were isolated from HIV-infected cART recipients with the EasySep Human Memory CD4^+^ T-cell enrichment kit (Stemcell). The purity of resting (CD69^neg^HLA-DR^neg^) and memory (CD45RA^neg^CD45RO^+^) CD4^+^ T cells was assessed by flow cytometry and was routinely >95%. Cells were rested overnight before use.

### In Vitro Resting CD4^+^ T-Cell Model of HIV-1 Latency

Resting CD4^+^ T cells isolated from HIV-seronegative individuals were plated at 4 × 10^6^ cells/mL in RP10 medium and treated with 100 nM CCL19 for 2 hours. Cells were then centrifuged and resuspended at 10 × 10^6^ cells/mL in RP10 medium. HIV_NL4.3_ (100 ng p24/1 × 10^6^ cells) was added, and spinoculation was performed at 1200×*g* for 120 minutes at room temperature. Cells were then washed 3 times with PBS, resuspended at 2 × 10^6^ cells/mL in RP10 medium with IL-2 (30 U/mL), and left in culture for 3 days. HIV-1 latency was confirmed by evaluating integrated HIV-1 DNA [23] and HIV-1 *gag* RNA [24] by polymerase chain reaction (PCR) analysis and evaluating p24 production by ELISA.

### MG1 Infection of Cell Lines and Primary Cells

Prior to MG1 infection, cell lines were passaged at 0.5 × 10^6^ cells/mL for 16–18 hours to allow entry into exponential growth phase. A total of 1 × 10^6^ cells were seeded in a 24-well plate at 5 × 10^6^ cells/mL in RP10 medium without phenol red indicator (ThermoFisher Technologies). Cell lines were then mock infected or infected with MG1 at a multiplicity of infection (MOI) of 0.00001–0.1 for 2 hours at 37°C, after which the medium volume was increased to maintain cells at a concentration of 1 × 10^6^ cells/mL. MG1 infection and cell viability were quantified 12–28 hours after infection.

Resting CD4^+^ T cells infected with HIV-1 in vitro and memory CD4^+^ T cells from patients were washed with PBS and plated in 24-well plates at a concentration of 5 × 10^6^ cells/mL in RP10 medium with IL-2 (30 U/mL) and RAL (10 μM). Cells were then mock infected or infected with MG1 at 10-fold serial dilutions (MOI, 0.1–10) for 2 hours at 37°C, after which the medium volume was increased to maintain cells at a concentration of 1 × 10^6^ cells/mL. MG1 infection and cell viability were quantified by flow cytometry 24 and 48 hours after infection. After 48 hours of MG1 infection, cells were washed twice in PBS, and cell pellets were stored at −80°C for quantification of integrated HIV-1 DNA or were prepared for viral outgrowth assay.

### Flow Cytometry

To evaluate purity, 1 × 10^5^ resting and memory CD4^+^ T cells were stained with anti–CD4-phycoerythrin-cyanin7 (clone SK3; BioLegend), anti–CD69-phycoerythrin (clone 298614; R&D systems), anti–HLA-DR-allophycocyanin (clone L243; BioLegend), and anti–CD45RO-phycoerythrin (clone UCHL1; BioLegend) antibodies. To evaluate low-density lipoprotein receptor (LDL-R) expression in cell lines, 1 × 10^5^ cells were stained using an anti–human LDL-R-PE antibody (clone 472413; R&D Systems). Nonspecific staining was monitored using isotype-matched control antibodies. Cells were fixed in 1% paraformaldehyde for 15 minutes prior to analysis using the FACSCalibur flow cytometer (BD Biosciences, Mississauga, Canada). As MG1 has been engineered to express enhanced GFP [15, 17], MG1 infection in cell lines and primary cells was quantified by GFP expression. In parallel, cell death was assessed by staining with propidium iodide (BioLegend) as per the manufacturer’s protocol.

### Viability Assay

At each time point of MG1 infection in cell lines, 1 × 10^5^ cells from each infection condition (MOI range, 0.00001–0.1 plaque-forming units/cell) were plated in 96-well plates in quadruplicate. AlamarBlue Cell Viability Reagent (ThermoFisher Scientific), diluted 1 in 5 in RP10 medium without phenol red indicator, was added to each well and incubated at 37°C for 4 hours. Fluorescence was read at an excitation wavelength of 530 nm and an emission wavelength of 590 nm, using the Fluoroskan Ascent Microplate Fluorometer (ThermoFisher Scientific).

### CellTrace Carboxyfluorescein Succinimidyl Ester (CFSE) Cell Proliferation Assay

Cell lines were plated at a concentration of 0.5 × 10^6^ cells/mL in RP10 medium for 16–18 hours. Cells were then counted and washed, and 1 × 10^6^ cells per condition were stained with 5 μM CFSE (Life Technologies) as indicated in the manufacturer’s instructions. Following CFSE staining, cells were plated at a concentration of 1 × 10^6^ cells/mL in serum-free RP10 medium or in RP10 medium with 0.25 μM colchicine (Sigma Aldrich). CFSE staining was evaluated at 0, 24, 48, and 72 hours by flow cytometry.

### Viral Outgrowth Assay

The viral outgrowth assay performed was adapted from previously established protocols [25, 26]. For the in vitro model of latency, 2.5 × 10^5^ resting CD4^+^ T cells were collected after 48 hours of MG1 infection and washed 5 times in PBS to remove any residual MG1. Cells were resuspended in RP10 medium with 5 μg/mL PHA and 30 U/mL IL-2 at a concentration of 0.5 × 10^6^ cells/mL and incubated at 37°C for 24 hours. PHA/IL-2–activated, CD8-depleted PBMCs were then added to the activated CD4^+^ T cells in 10-fold excess at a concentration of 5 × 10^6^ cells/mL in RP10 medium with IL-2. Culture supernatants were collected on days 0, 4, 7, 10, and 14, and viral outgrowth was evaluated by p24 ELISA.

CD45RO^+^ memory CD4^+^ T cells from cART recipients were infected with MG1 for 48 hours, after which 1 × 10^6^ cells were collected and washed 5 times in PBS. Cells were resuspended at a concentration of 1 × 10^6^ cells/mL in RP10 medium with 30 U/mL IL-2, plated in 24-well plates precoated with 1 μg/mL anti-CD3 and anti-CD28, and incubated at 37°C for 24 hours. PHA/IL-2–activated CD4^+^ T cells were added as feeder cells in 5-fold excess at a concentration of 5 × 10^6^ cells/mL in RP10 medium with IL-2. A second round of feeder cells were added in a similar manner at day 7 of viral outgrowth, and cells were incubated for a total of 21 days. Culture supernatant was collected on days 0, 4, 7, 10, 14, and 21, and viral outgrowth was evaluated by p24 ELISA and HIV-1 *gag* RNA PCR.

### p24 ELISA

Quantification of p24 antigen was performed by ELISA. Cell-free supernatants were lysed with 1% Triton-X, and p24 antigen expression was quantified by the HIV-1 p24 Antigen Capture Kit (Frederick National Laboratory for Cancer Research and NIH AIDS Reagent Program) following the manufacturer’s protocol.

### RNA and DNA Extraction

Cell-associated RNA was extracted using the Illustra RNAspin mini kit (GE Healthcare Life Sciences, Mississauga) according to the manufacturer’s instructions. Cell-free RNA was extracted from 180 μL of supernatant, using the QIAmp viral RNA mini kit (Qiagen, Venlo, the Netherlands). Genomic DNA extraction for the quantification of integrated HIV-1 DNA was performed as described elsewhere [23]. Briefly, cells were digested in lysis buffer (10 mM Tris-HCl [pH 8.0], 50 nM KCl, and 400 μg/mL proteinase K; Invitrogen, Burlington, Canada) for 12–16 hours at 55°C with shaking. RNA and DNA integrity was monitored by agarose gel electrophoresis, and concentrations were measured using the ND-1000 Spectrophotometer (NanoDrop, Wilmington, DE). All samples were stored at −20°C.

### Reverse Transcription (RT)–qPCR Analysis of Cell-Associated and Cell-Free RNA

RT-qPCR reactions were performed using the iScript cDNA Synthesis Kit (BioRad, Hercules, CA) according to the manufacturer’s protocol. Isolated cDNA was evaluated for HIV-1 using the primer-probe set targeting a conserved region of *gag* as previously described [24, 27]. RT-PCR reaction was performed using the SsoAdvanced Universal Probes Supermix (BioRad) on the CFX Connect Real-Time PCR Detection System (BioRad).

### Quantification of Integration Events

A 2-step, nested PCR quantifying integrated HIV-1 DNA and CD3 was performed with previously described primer-probe sets [23]. In addition, digital droplet PCR (ddPCR) was performed using the QX200 Droplet Digital PCR system (BioRad) with the ddPCR Supermix for Probes (No dUTP) and the first-step PCR product obtained from the nested PCR described above. The reaction mix was aerosolized using the QX200 Droplet Generator (BioRad) as per the manufacturer’s instructions. PCR reactions were then carried out using the C1000 Touch Thermal Cycler (BioRad), with the following amplification steps for all reactions: denaturation (95°C for 10 minutes), 40 cycles of amplification (95°C for 30 seconds and 57°C for 1 minute), and droplet stabilization (4°C for 5 minutes and 90°C for 5 minutes). Absolute quantification of integrated HIV-1 and CD3 DNA was performed using the QX200 Droplet Reader (BioRad).

### Statistical Analysis

Statistical analyses were performed using Prism software (GraphPad), and *P* values of ≤.05 were considered significant. As determined a priori, comparisons of MG1 infection between cell lines, as well as mock-infected cells and cells latently infected with HIV-1 in vitro, were performed by 2-way analysis of variance (ANOVA) with the Bonferroni correction for post hoc analysis. The effect of MG1 infection on integrated DNA and viral outgrowth was evaluated by Kruskal-Wallis 1-way ANOVA on ranks with the Dunn multiple comparison test or by 1-way ANOVA with the Dunnett multiple comparison test, as well as by the paired *t* test.

### Study Approval

Volunteers with and without HIV-1 infection provided written informed consent. This study was approved by the Ottawa Health Science Network Research Ethics Board.

## RESULTS

**Figure 1.**
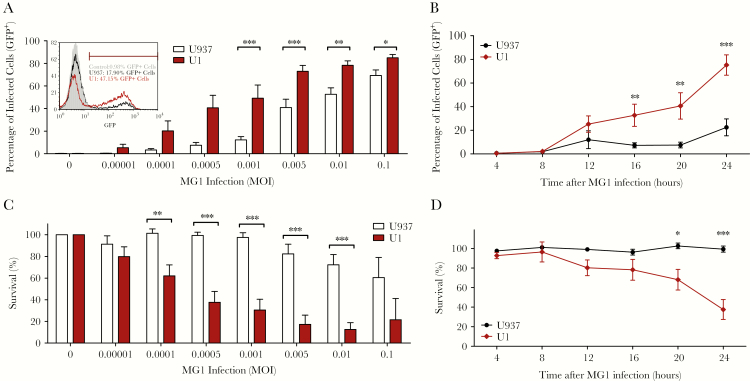
MG1 preferentially infects and eliminates cell lines latently infected with human immunodeficiency virus type 1 (HIV-1). *A*, The percentage of green fluorescent protein (GFP)–expressing (GFP+) cells 20 hours after MG1 infection, by flow cytometry (n = 8). Inset representative histograms show MG1 infection (GFP+ cells) of U1 cells latently infected with HIV and uninfected U937 cells 20 hours after MG1 infection. *B*, Percentage GFP+ cells following infection (multiplicity of infection [MOI], 0.005) at the indicated time points (n = 8). *C*, The percentage of viable cells 20 hours after MG1 infection, by the AlamarBlue assay (n = 8). *D*, The percentage of viable cells following MG1 infection (MOI, 0.005) over time (n = 8). *P < .05; **P < .01, and ***P < .001 by 2-way analysis of variance with the Bonferroni post hoc test for multiple comparisons between U937 and U1 cells. Data represent mean values ± standard errors of the mean; n values represent separate biological replicates.

Impaired IFN-I signaling was previously demonstrated in the latently HIV-1 infected cell lines, U1 and OM10.1 [9]. We therefore began by assessing the ability of MG1 to infect and eliminate U1 and OM10.1 cells, in comparison with their respective HIV-uninfected parental cell lines, U937 and HL60. While both U1 and U937 cells were infected with the GFP-expressing MG1, U1 cells were significantly more susceptible to infection (Figure 1a, b), as were OM10.1 cells, relative to HL60 cells (Supplementary Figure 1a,b). In parallel, significantly greater MG1-mediated eradication of U1 cells, relative to U937 cells (Figure 1c, d), and of OM10.1 cells, relative to HL60 cells (Supplementary Figure 1c,d), was observed. Thus, cell lines latently infected with HIV are preferentially infected and killed by MG1, relative to their uninfected parental cells.

**Figure 2.**
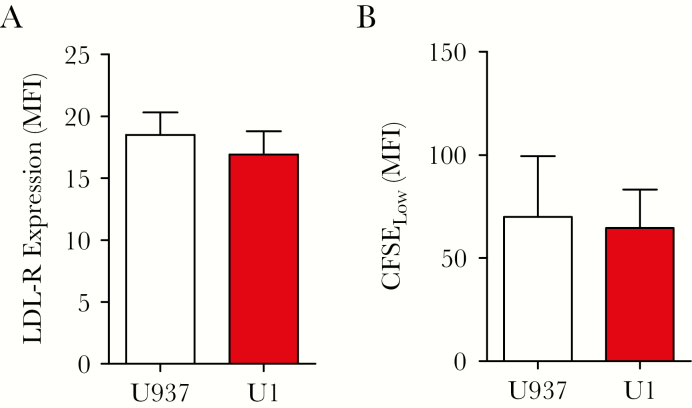
Investigation of potential cell-intrinsic mechanisms facilitating MG1 infection of U937 and U1 cells. *A*, Surface expression of low-density lipoprotein receptor (LDL-R), by flow cytometry (n = 5). *B*, Intensity of carboxyfluorescein succinimidyl ester (CFSE) dilution after 24 hours of culture, by flow cytometry (n = 3). n values represent separate biological replicates. MFI, mean fluorescence intensity.

Next, potential cell-intrinsic mechanisms underlying enhanced MG1 infection were investigated. MG1 binding and entry into HIV-1–infected cells was assessed by measuring the expression of LDL-R, the primary receptor for Maraba virus [28–30]. LDL-R expression was similar between U1 and U937 cells (Figure 2a) and between HL60 and OM10.1 cells (Supplementary Figure 2a). Entry into cell cycle and rate of cellular proliferation have also been identified as critical determinants of rhabdovirus replication [31]. Proliferation was not different between U1 and U937 cells (Figure 2b) or between HL60 and OM10.1 cells (Supplementary Figure 2b). Therefore, enhanced infection and elimination of cell lines with latent HIV-1 infection could feasibly be attributed to IFN-I signaling defects.

**Figure 3.**
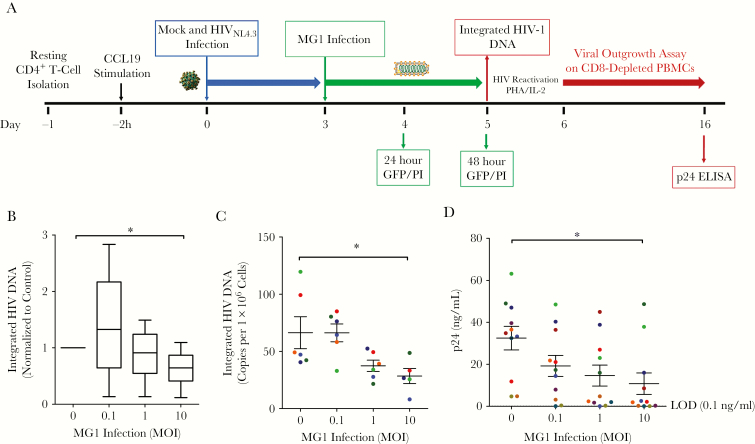
MG1 infection of resting CD4^+^ T cells latently infected with human immunodeficiency virus type 1 (HIV-1) results in the elimination of replication-competent HIV-1. *A*, Experimental design for MG1 infection of an in vitro primary cell model of HIV-1 latency in resting CD4^+^ T cells. *B*, Integrated HIV-1 DNA level 48 hours after MG1 infection (n = 12) relative to that in MG1-uninfected controls, measured by quantitative polymerase chain reaction analysis. *P = .0086 by 1-way analysis of variance (ANOVA) on ranks, and *P* < .05 by the Dunn multiple comparison test. *C*, Copies of integrated HIV-1 DNA per 10^6^ cells 48 hours after MG1 infection (n = 6), measured by digital droplet PCR. *P = .0162 by 1-way ANOVA on ranks, and *P* < .05 by the Dunn multiple comparison test. *D*, Concentration of p24 antigen in supernatants at day 10 of a viral outgrowth assay (n = 9). *P = .0288 by 1-way ANOVA, and *P* < .05 by the Dunnett multiple comparison test. Data points in panel *D* are the average of duplicate technical replicates. Data represent mean values ± standard errors of the mean; n values represent separate biological replicates. Individual donors are defined by color in panels *C* and *D*. ELISA, enzyme-linked immunosorbent assay; GFP, green fluorescent protein; MOI, multiplicity of infection; PBMC, peripheral blood mononuclear cell.

MG1 infection in primary cells latently infected with HIV-1 was investigated using a resting CD4^+^ T-cell model of latency [32], as outlined in Figure 3a. As expected, only a small proportion of cells carried integrated DNA 3 days after infection (Supplementary Figure 3a). Viral replication, as measured by cell-associated HIV-1 *gag* RNA, was absent at baseline and required PHA/IL-2 stimulation, confirming a state of latency (Supplementary Figure 3b). Using this model, mock-infected cells and cells latently infected with HIV-1 were infected with MG1 for 48 hours. Negligible differences in MG1 infection were observed between cells with and those without HIV-1 infection, even at the highest MOI (Supplementary Figure 4a). Importantly, minimal MG1-mediated cell death was observed in primary cells with and those without HIV-1 infection (Supplementary Figure 4b). As cells latently infected with HIV-1 represent <1% of the total cell population in this model, the absence of observable differences was to be expected. Moreover, the lack of killing of healthy primary cells was reassuring with respect to the safety of MG1 as a potential therapeutic agent.

Next, the ability of MG1 to eliminate resting CD4^+^ T cells latently infected with HIV-1, which would lead to a decrease in integrated HIV-1 DNA and levels of inducible HIV-1, was evaluated. As hypothesized, a significant decrease in integrated DNA was demonstrated 48 hours after MG1 infection, relative to MG1-uninfected controls (Figure 3b and Supplementary Figure 5). These data were confirmed using ultrasensitive ddPCR, allowing low copy numbers of HIV DNA to be measured quantitatively as the number of copies per 1 × 10^6^ cells (Figure 3c). Stimulation of cells in a modified viral outgrowth assay [25] resulted in an MOI-dependent decrease in HIV-1 p24 antigen expression (Figure 3d), paralleling the observed decrease in integrated HIV-1 DNA.

**Figure 4.**
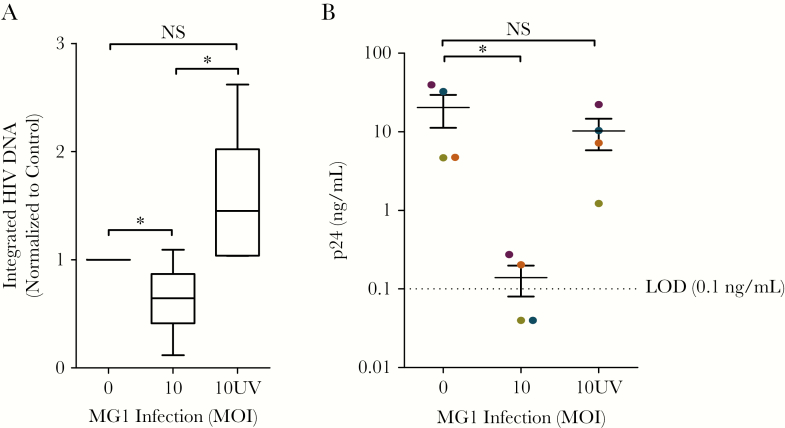
Elimination of replication-competent human immunodeficiency virus type 1 (HIV-1) in HIV-infected resting CD4^+^ T cells is prevented by UV light inactivation of MG1. *A*, Integrated HIV-1 DNA 48 hours after infection with MG1 that was or was not inactivated by UV light (n = ≥6). *P < .0001 by 1-way analysis of variance (ANOVA) on ranks, and *P* < .05 by the Dunn multiple comparison test. *B*, p24 concentration at day 10 of a viral outgrowth assay following infection with MG1 or UV light–inactivated MG1 (n = 4). *P = .0228 by 1-way ANOVA on ranks, and *P* < .05 by the Dunn multiple comparison test. Data points in panel *B* are the average of duplicate technical replicates. Data represent mean values ± standard errors of the mean; n values represent separate biological replicates. Individual donors are defined by color in panel *B*. MOI, multiplicity of infection; NS, not significant.

To investigate if this observation was dependent on the ability of MG1 to infect and replicate within CD4^+^ T cells latently infected with HIV-1, MG1 was UV inactivated prior to infection. Although UV light–inactivated rhabdoviruses that lack the capacity to replicate can trigger the immunogenic apoptosis of cancer cells [33], exposure of CD4^+^ T cells latently infected with HIV to UV light–inactivated MG1 had no effect on HIV-1 DNA or outgrowth (Figure 4a, b).

**Figure 5.**
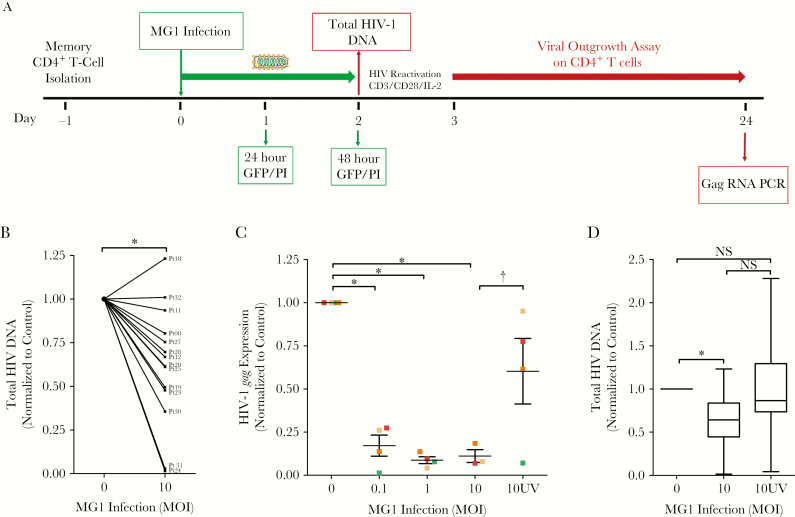
MG1 infection of memory CD4^+^ T cells from combination antiretroviral therapy recipients with virologically suppressed human immunodeficiency virus type 1 (HIV-1) infection results in a reduction in the size of the inducible reservoir. *A*, Experimental design for the MG1 infection of memory CD4^+^ T cells isolated from HIV-1–infected individuals. *B*, Cell-associated total HIV-1 DNA level 48 hours after MG1 infection in each HIV-1*–*infected individual, relative to MG1-uninfected controls (n = 14). *P = .0011 by the paired *t* test. *C*, Relative HIV-1 *gag* level in supernatant after 21 days of viral outgrowth (n = 4). *P < .001 by 1-way analysis of variance (ANOVA), and *P* < .05 by the Dunnett multiple comparison test; ^†^*P* = .0354 by paired t test for comparison of the multiplicity of infection (MOI) 10 group to the 10 UV group. *D*, Cell-associated total HIV-1 DNA level 48 hours after MG1 infection, relative to MG1-uninfected controls (n = 13). *P = .0035 by 1-way ANOVA on ranks, and *P* < .05 by the Dunn multiple comparison test. Data represent mean values ± standard errors of the mean; n values represent separate biological replicates. Individual HIV-infected donors are defined by color in panels *C* and *D*. ELISA, enzyme-linked immunosorbent assay; GFP, green fluorescent protein; NS, not significant; PCR, polymerase chain reaction.

MG1-mediated elimination of cells latently infected with HIV-1 was finally assessed ex vivo, using memory (CD45RO^+^) CD4^+^ T cells isolated from effectively treated HIV-infected individuals (Figure 5a). This population, which is composed of central memory, transitional memory, and effector memory subsets, was assessed because it is believed to harbor the majority of proviral HIV-1 DNA [34]. As before, low frequencies of MG1 infection with no appreciable increase in cell death were observed 48 hours after infection (Supplementary Figure 6a,b). However, a significant decrease in cell-associated HIV-1 DNA was observed in cells from 12 of 14 individuals (Figure 5b).

Finally, the effect of MG1 on replication-competent HIV-1 was evaluated by a viral outgrowth assay [25]. MG1 infection resulted in a significant decrease in HIV-1 *gag* RNA expression in cell-free supernatants at day 21 of outgrowth (Figure 5c), with an approximately 90% decrease in gag RNA expression at MG1 MOIs of 1 and 10. Exposure to UV light–inactivated MG1 had no significant effect (Figure 5c, d).

## DISCUSSION

HIV-1 cure strategies have largely focused on the shock and kill approach, which aims to reverse viral latency and facilitate immune-mediated eradication of HIV-1–infected cells. This approach has, however, not yet been proven effective in clinical settings. Alternatively, latently infected cells could be targeted on the basis of the expression of a surface marker unique to that population. CD32a was recently identified as one such marker but appears to identify only approximately 50% of the CD4+ T-cell reservoir, suggesting that its usefulness in the eradication of latently infected cells may be limited [35].

A third option would be to target an intracellular defect that arises as a result of HIV-1 infection and is therefore present in all infected cells. Defective IFN-I signaling and antiviral IFN-I–stimulated protein expression has been observed during productive HIV-1 infection of primary cells [36–40], as well as in latently infected cell lines, and may therefore serve as a useful target to differentiate HIV-infected cells from uninfected cells. It is unknown whether defective IFN-I signaling during HIV-1 latency results from an irreversible process occurring during initial infection or reflects the effects of ongoing, low-level viral replication. Regardless, targeting an intracellular defect unique to HIV-1–infected cells using the oncolytic rhabdovirus MG1 may be a viable approach to clearing the latent reservoir, because it does not require the complete reactivation of latent provirus, nor does it appear to require the expression of specific cellular proteins to identify target cells.

MG1-mediated killing was demonstrated using U1 and OM10.1 cell lines latently infected with HIV, which are known to possess IFN-I signaling defects [9]. MG1 also reduced replication competent HIV-1 in a primary CD4^+^ T-cell model of latency and in memory CD4^+^ T cells obtained from HIV-1–infected individuals, although it has not been definitively shown that impaired IFN-I signaling is the explanation for these findings. Exploiting the IFN-I response to eliminate cells latently infected with HIV-1 has previously been considered by others. Li et al recently found that the retinoic acid derivative acitretin induced apoptosis in cells latently infected with HIV-1 by activating retinoic inducible gene I [8].

Although it would be desirable to measure selective MG1 infection of latently infected primary CD4^+^ T cells, these cells are present at very low frequencies in appropriate in vitro models [41–43] and are virtually indistinguishable from their uninfected counterparts. The observed decreases in integrated viral DNA and inducible HIV-1, without nonspecific cell death in the primary CD4^+^ T-cell population, were therefore interpreted as the elimination of latently infected cells. This approach is consistent with previous studies that investigated the fate of the latent HIV-1 reservoir following pharmacological intervention [44–46].

 The ability of MG1 to selectively kill IFN-defective cancer cells has proven feasible in animal models and is now being studied in patients with metastatic solid tumors and non–small-cell lung cancer (clinical trials identifiers NCT02285816 and NCT02879760). This has laid the groundwork for MG1 therapy in other contexts, including HIV-1 infection. While further refinement may be achieved by enhancing MG1 specificity, these findings, along with the safety data being acquired, suggest that a proof-of-principle clinical trial in HIV-infected individuals is currently feasible.

## Supplementary Data

Supplementary materials are available at *The Journal of Infectious Diseases* online. Consisting of data provided by the authors to benefit the reader, the posted materials are not copyedited and are the sole responsibility of the authors, so questions or comments should be addressed to the corresponding author.

Supplementary Figure 1

Supplementary Figure 2

Supplementary Figure 3

Supplementary Figure 4

Supplementary Figure 5

Supplementary Figure 6

## Notes

***Acknowledgments.*** We thank the study participants, without whom this research would not be possible; the National Institutes of Health (NIH) AIDS Reagent Program, Division of AIDS, National Institute of Allergy and Infectious Diseases, NIH, for cell lines, plasmid, and reagents; and Dr John Bell and Dr Dave Stojdl, for their generous contribution of MG1.

***Financial support.*** This work was supported by the Department of Medicine, University of Ottawa; the Canadian HIV Cure Enterprise Team (grant HIG-133050 [from the Canadian Institutes of Health Research {CIHR} in partnership with Canadian Foundation for AIDS Research and the International AIDS Society] to J. B. A.); Vanier Canada Graduate Scholarships (to N. R.); and the CIHR (doctoral research award to T. S. S.).

***Potential conflicts of interest.*** All authors: No reported conflicts of interest. All authors have submitted the ICMJE Form for Disclosure of Potential Conflicts of Interest. Conflicts that the editors consider relevant to the content of the manuscript have been disclosed.

Presented in part: 25th Annual Canadian Conference on HIV/AIDS Research, Winnipeg, Canada, May 2016; Ottawa Hospital Research Institute 2015 Research Meeting, Ottawa, Canada, May 2015; 8th International AIDS Society: Towards a HIV Cure Symposium, Vancouver, Canada, July 2015; 24th Annual Canadian Conference on HIV/AIDS Research, Toronto, Canada, May 2015; Mechanisms of HIV Persistence: Implications for a Cure, Boston, Massachusetts, April 2015; 23rd Annual Canadian Conference on HIV/AIDS Research, St. John’s, Newfoundland, Canada, May 2014.
