# Supplementary figures and images for: The Oncolytic Virus MG1 Targets and Eliminates Cells Latently Infected With HIV-1: Implications for an HIV Cure
Source: J Infect Dis. 2017 Dec 8;217(5):721–30. doi: 10.1093/infdis/jix639 (PMC5853232; doi:10.1093/infdis/jix639)

## Slide 1
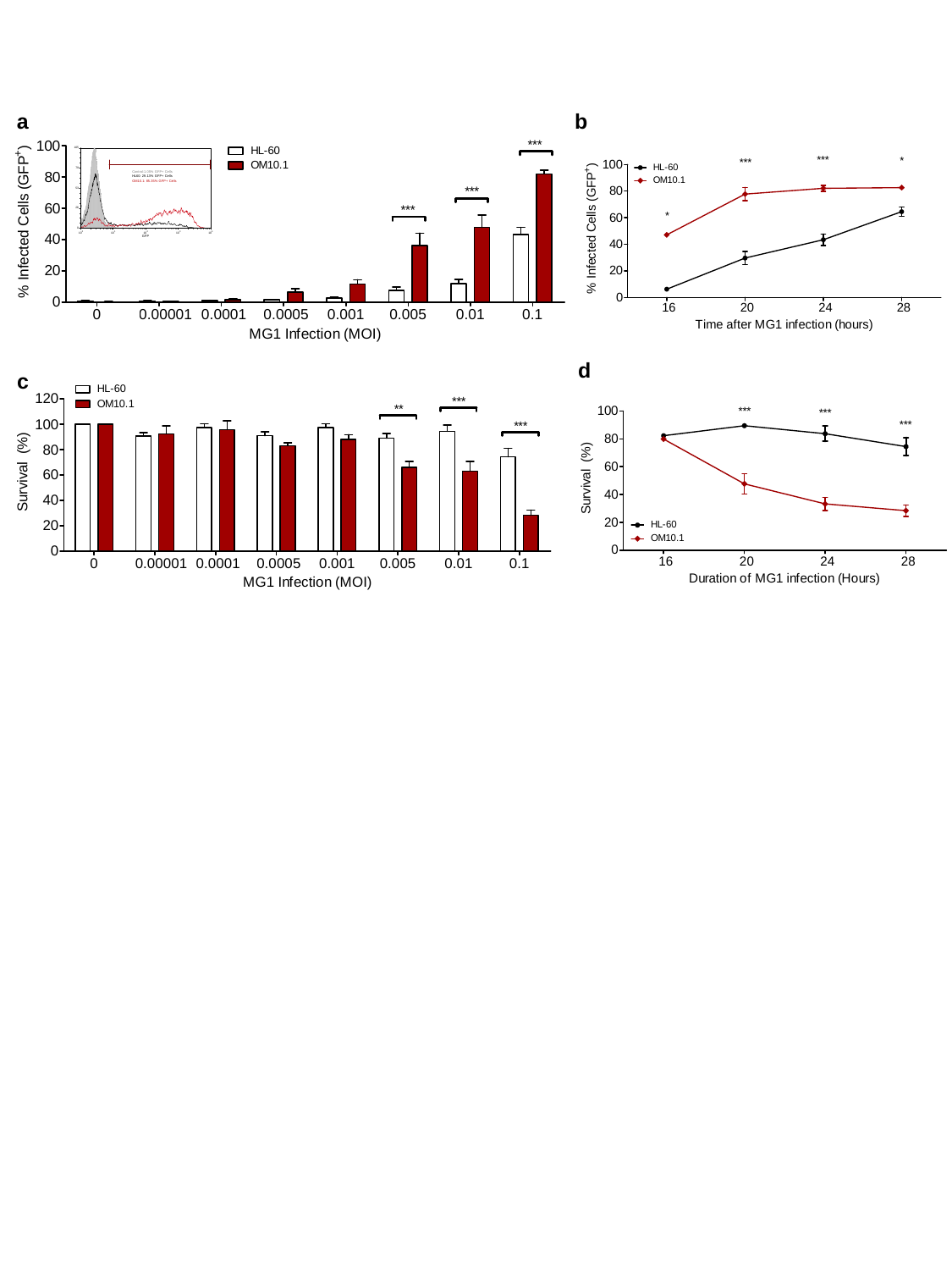

a
b
d
c

Supplement: Supplementary Figure 1 [file jix639_suppl_figure1.pptx]

## Slide 1
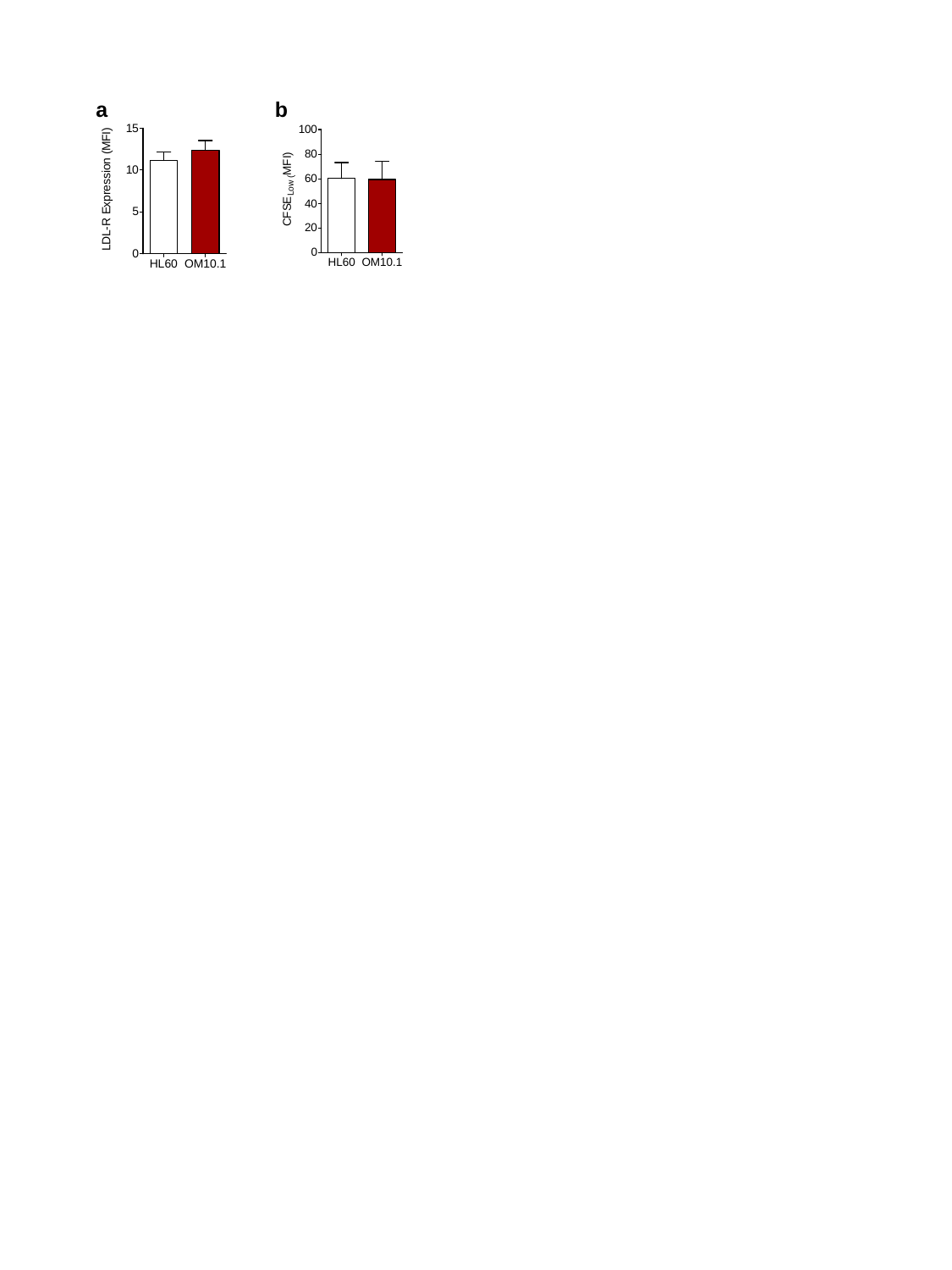

a
b

Supplement: Supplementary Figure 2 [file jix639_suppl_figure2.pptx]

## Slide 1
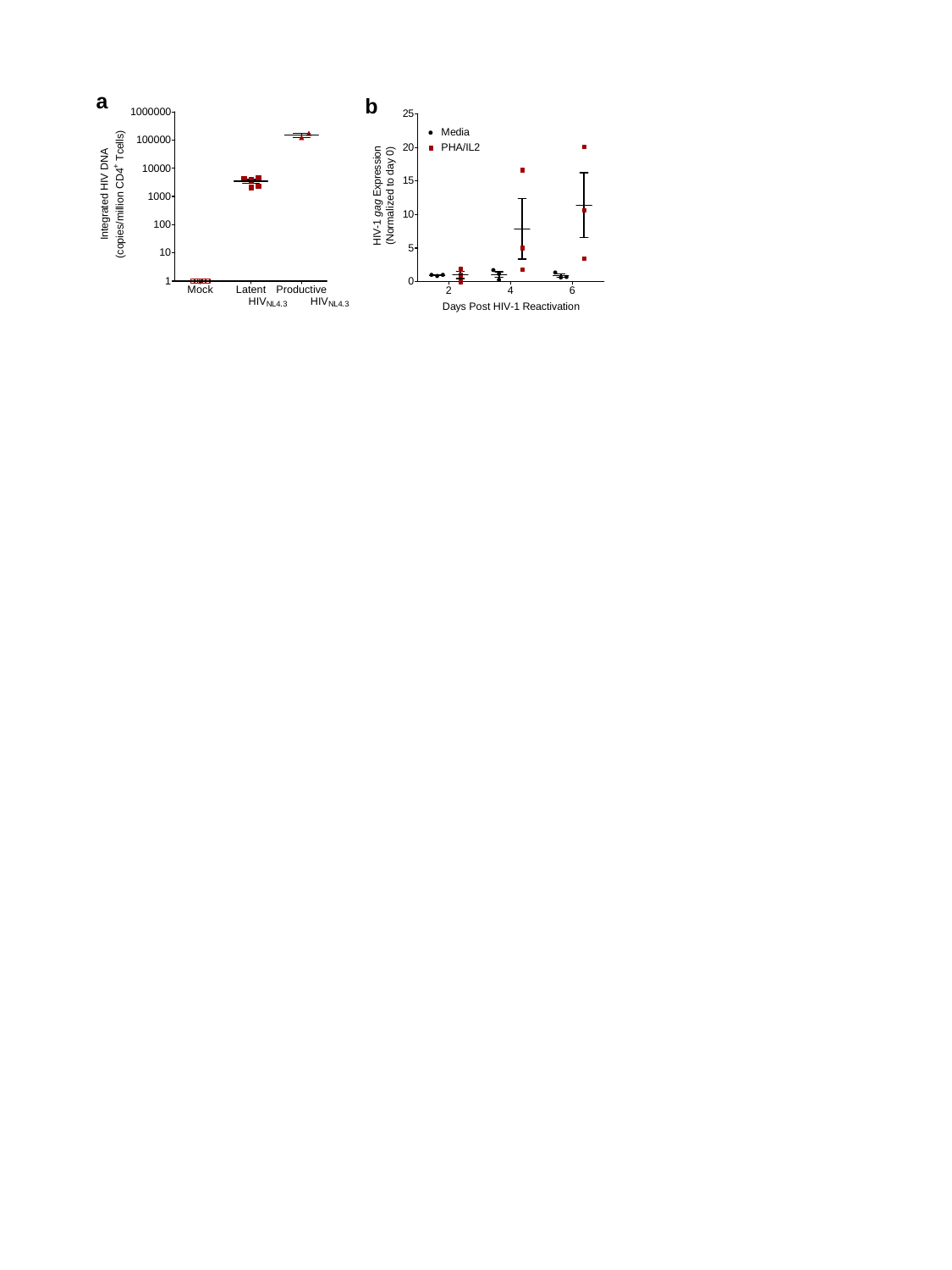

a
b

Supplement: Supplementary Figure 3 [file jix639_suppl_figure3.pptx]

## Slide 1
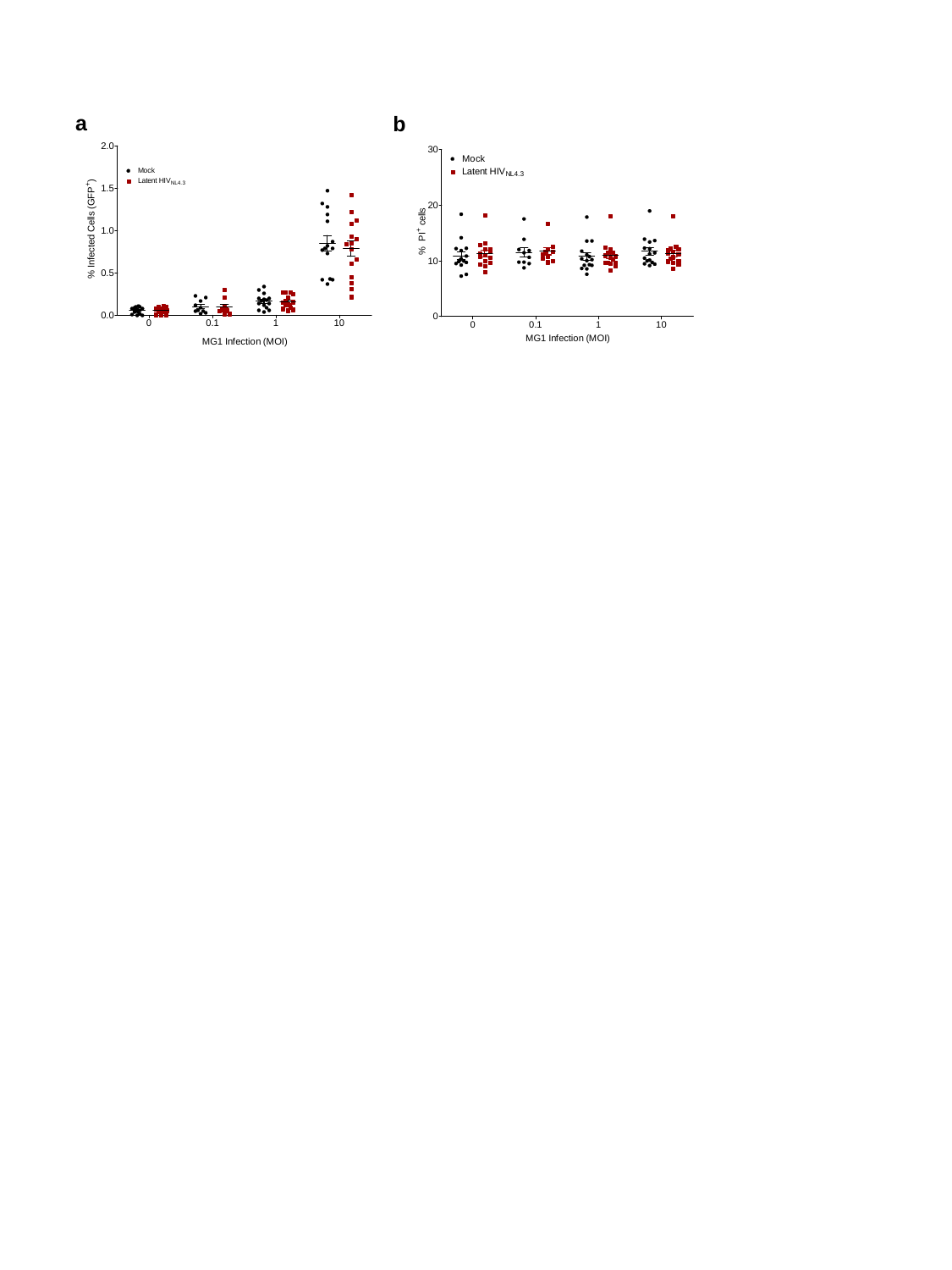

a
b

Supplement: Supplementary Figure 4 [file jix639_suppl_figure4.pptx]

## Slide 1
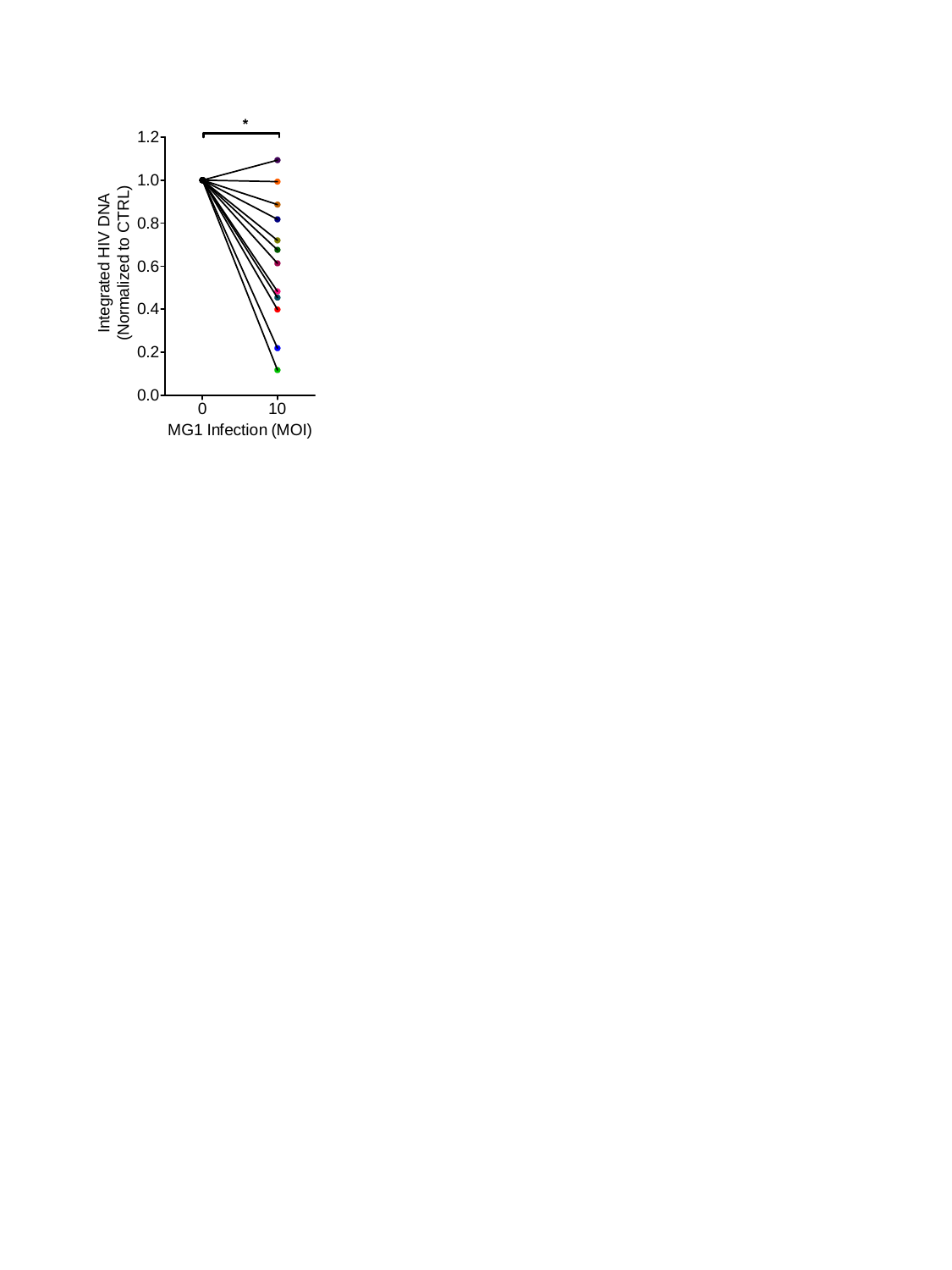

Supplement: Supplementary Figure 5 [file jix639_suppl_figure5.pptx]

## Slide 1
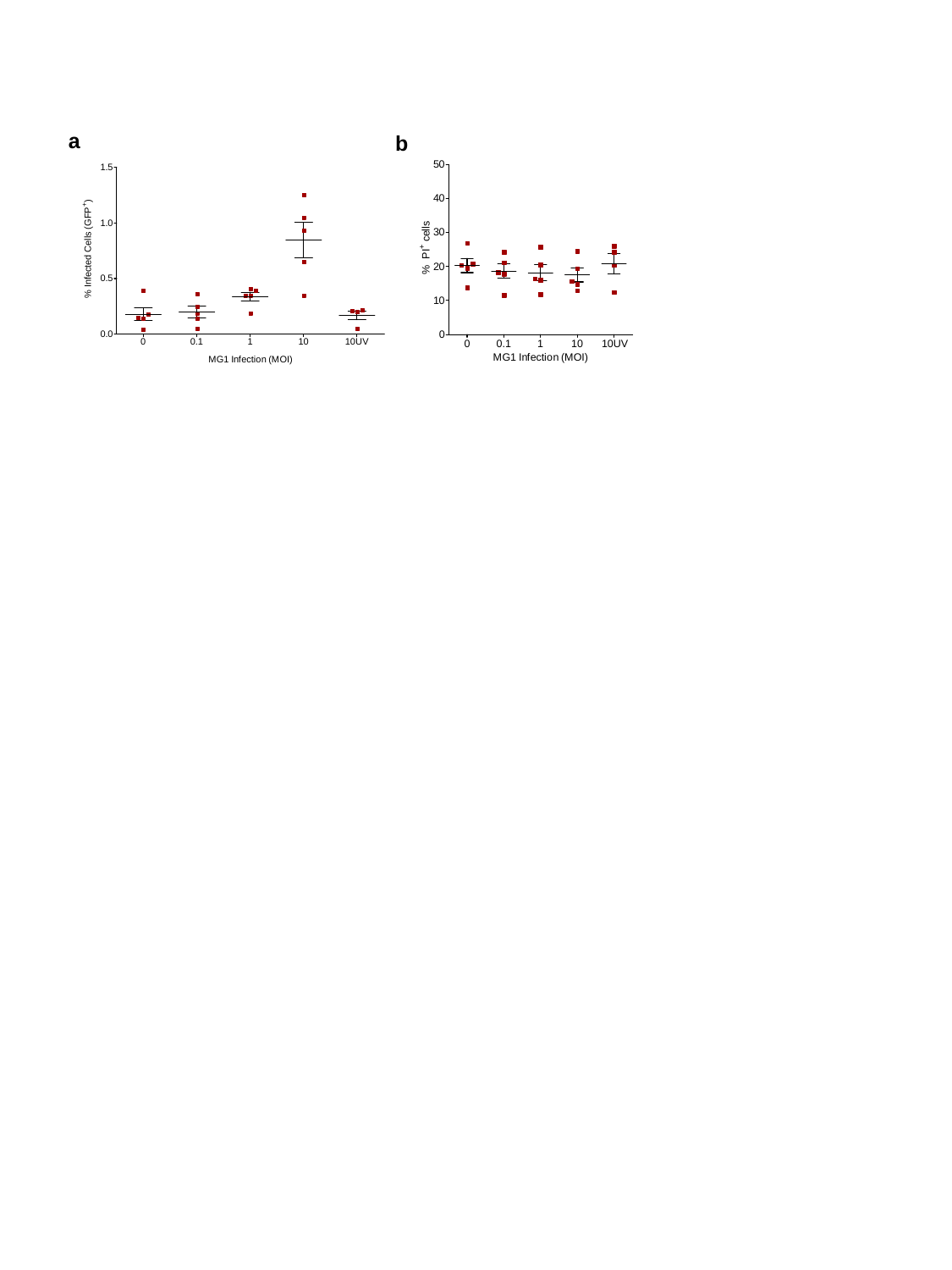

a
b

Supplement: Supplementary Figure 6 [file jix639_suppl_figure6.pptx]
